# Impact of Color Space and Color Resolution on Vehicle Recognition Models

**DOI:** 10.3390/jimaging10070155

**Published:** 2024-06-26

**Authors:** Sally Ghanem, John H. Holliman

**Affiliations:** Oak Ridge National Laboratory, Oak Ridge, TN 37830, USA; hollimanjh@ornl.gov

**Keywords:** vehicle recognition, color space, machine learning, optimization, traffic monitoring, surveillance systems

## Abstract

In this study, we analyze both linear and nonlinear color mappings by training on versions of a curated dataset collected in a controlled campus environment. We experiment with color space and color resolution to assess model performance in vehicle recognition tasks. Color encodings can be designed in principle to highlight certain vehicle characteristics or compensate for lighting differences when assessing potential matches to previously encountered objects. The dataset used in this work includes imagery gathered under diverse environmental conditions, including daytime and nighttime lighting. Experimental results inform expectations for possible improvements with automatic color space selection through feature learning. Moreover, we find there is only a gradual decrease in model performance with degraded color resolution, which suggests the need for simplified data collection and processing. By focusing on the most critical features, we could see improved model generalization and robustness, as the model becomes less prone to overfitting to noise or irrelevant details in the data. Such a reduction in resolution will lower computational complexity, leading to quicker training and inference times.

## 1. Introduction

Vehicle recognition technology impacts a wide range of applications, from enhancing security measures to improving traffic management and providing insights for business strategies. Some applications need recognition at the basic level of classification, while others demand a more advanced level of re-identification. Vehicle classification, the more abstract operation, categorizes vehicles based on make and model and possibly other parameters such as color, model year, or trim level. On the other hand, vehicle re-identification achieves a higher level of precision, identifying a specific vehicle as one that was previously observed. Insurance and traffic flow analyses, for example, stand to benefit even from broad categorizations, whereas toll collection requires precise vehicle identification.

The development of models for vehicle recognition generally aims to push capabilities as close toward re-identification as possible, because such models support more applications. Certain use cases, though, concern vehicle targets that do not cooperate in being identified. These vehicles will not, for instance, carry transponders in support of electronic toll collection. Moreover, license plate information in such cases must be assumed unreliable or unavailable. As an example, in the realm of security, surveillance, or law enforcement, one cannot discount deliberate deception by the vehicle operator. Plates can be obscured, blocked, removed, or switched with those from another vehicle. In other applications, plate reading may not be practical or even permissible: Motorists entering toll roads and controlled parking implicitly agree to the capture of identifying information, but collection in other contexts may violate privacy conventions. While possibly helpful in establishing ground truth for training, a license tag has no role in inference within these applications.

With absent features such as plates that offer a one-to-one mapping to specific objects, the task of vehicle recognition becomes more challenging. Indeed, there are many similar-looking vehicles in circulation. In light of such ambiguities, models rely on vehicle features such as wheel designs and body contours. In [1], the authors proposed a part-regularized discriminative feature-preserving method to enhance the ability to perceive subtle discrepancies. Their approach employed three vehicle parts for detection, including the front light, back light, front window, back window, and vehicle brand. An end-to-end RNN-based hierarchical attention (RNN-HA) classification model for vehicle re-identification was introduced in [2]. The proposed RNN-based module models can effectively capture subtle visual appearance cues, such as paint and windshield stickers. In [3], a local feature-aware model for vehicle re-identification was proposed to focus on learning discriminating parts that differ among vehicles. However, their model did not perform well under dim illumination conditions. In [4], a co-occurrence attention network (CAN) was introduced to extract consistent global features and local details with viewpoint information. Their model was trained by partition-and-reunion-based loss to narrow the intra-class distance and increase the inter-class distance. Color also plays an important role, being represented mathematically in a multidimensional color space, where each dimension corresponds to a color component. The choice of color space has potentially important ramifications for the accuracy and robustness of vehicle recognition algorithms based on digital images. Traditional color spaces like RGB, native to optical cameras, exhibit both strengths and weaknesses in facilitating vehicle recognition across diverse lighting conditions. The current work was commissioned to evaluate the effect of RGB transformations on model performance.

Prior research on color spaces (e.g., HSV [5], LAB [6], YCbCr [7]) demonstrates the influence of color space selection on the performance of recognition models, including in the context of vehicle recognition [8,9]. This body of work includes both simple linear transformations [10] and feature synthesis. Feature synthesis, by creating complex features through nonlinear operations, enables the capture of intricate relationships within the data that linear transformations might miss, thereby enhancing model performance by leveraging a more sophisticated understanding of data patterns [11,12]. Advances in machine learning, particularly through deep learning techniques [13,14] and the integration of domain knowledge [15], have also proved useful in optimizing color space selection to improve model performance. A study of the differential effects of various color spaces on convolutional neural networks (CNNs) [16] identifies the LUV color space as a viable alternative to RGB in achieving comparable results on the CIFAR10 dataset. Furthermore, research into feeding multiple color spaces into individual dense networks [17] found that certain color spaces more effectively represent specific classes. Results of this work suggest that the strategic choice and combination of color spaces might substantially influence the efficacy of vehicle recognition models, as well.

Despite their successes in image processing and computer vision, common color spaces have constraints that can limit their effectiveness at vehicle recognition. First, sensitivity to lighting changes can lead to significant variations in color representation, reducing recognition accuracy under variable outdoor conditions. Second, high correlation among channels can make it hard to isolate specific color information, negatively impacting the performance of recognition algorithms. Third, mixing of color and intensity information makes channel values more dependent on lighting and shadows. The lack of an invariant quantity in turn makes a recognition algorithm more sensitive to environmental factors and reduces its accuracy. Finally, non-uniform perceptual color representation can make recognition algorithms overly sensitive to minor, perceptually insignificant color changes, complicating algorithms and reducing performance. As RGB suffers many of these drawbacks, we explore alternative color spaces in Section 2. We will investigate the role of color spaces in distinguishing vehicles, identifying unique features, and ensuring robustness under different lighting conditions. In addition, we will evaluate other representations with progressively reduced color resolution, extending to the level of binary imagery. Our proposed approach will aid in narrowing down the input variables to identify those with the highest predictive value, demonstrating the importance of judicious feature selection in enhancing model efficiency and accuracy. Our objective is to investigate further simplifications of visual sensors and network architecture and more efficient training given the reduced information content being processed. The potential gains in processing efficiency from reducing color depth can be substantial. By focusing on the most critical features, we can also observe improved model generalization and robustness, as the model becomes less prone to overfitting to noise or irrelevant details in the data. An additional contribution of our work is the emergence of a hypothesis for improving model accuracies.

## 2. Experimental Setup

Here, we define some alternatives to RGB and discuss their properties that are relevant to vehicle recognition. Our investigation focuses on understanding the pivotal role of color spaces in matching different vehicles. In addition, we will evaluate other lower-resolution representations. Each color space and resolution corresponds to a set of experiments in model training and use. The subsequent analysis aims for an understanding of how each color space or resolution performs under diverse environmental conditions, so that specific characteristics that improve vehicle recognition might be identified.

Due to computing constraints, we choose specific color spaces from the multitude of options [18] that satisfy at least one of the following criteria: (1) The color space is commonly used in computational studies, irrespective of demonstrated utility in object recognition; (2) Previous work has shown the color space to have particular promise in mitigating confusion among similar images; (3) Color mapping allows us to explore a part of a computational space that may reveal new information relevant to training for vehicle re-identification. In the following, we will describe the adopted color transformations.

### 2.1. Conversion among Color Spaces

#### 2.1.1. Native RGB

**Figure 1:**
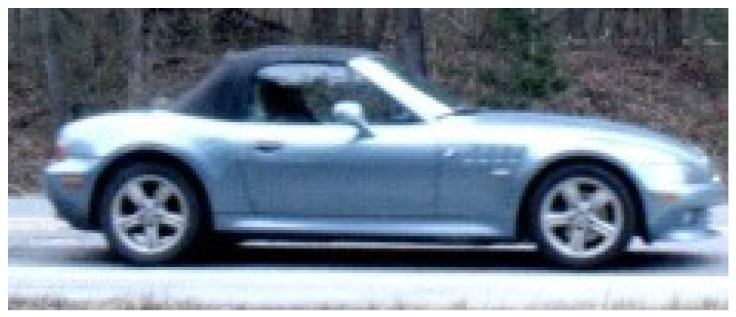
RGB.

The Blackfly® series camera, model BFLY-U3-13S2C-CS, collected daytime images in our study. This device features a 1.3 MP Sony IMX035 color CMOS sensor and captures images with a resolution of 1328 × 1048 pixels. Specifications regarding the camera’s spectral performance are available in a technical reference by FLIR Systems [19]. We captured night-time images with Sony camera model UMC-S3CA. This instrument has a 35 mm Exmor sensor with exceptional sensitivity in low-light conditions. In Figure 1, we show an example for an RGB image from the collected dataset.

#### 2.1.2. HSV

**Figure 2:**
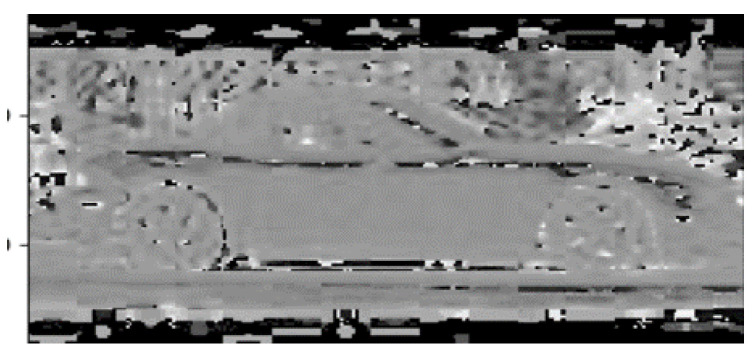
H.

**Figure 3:**
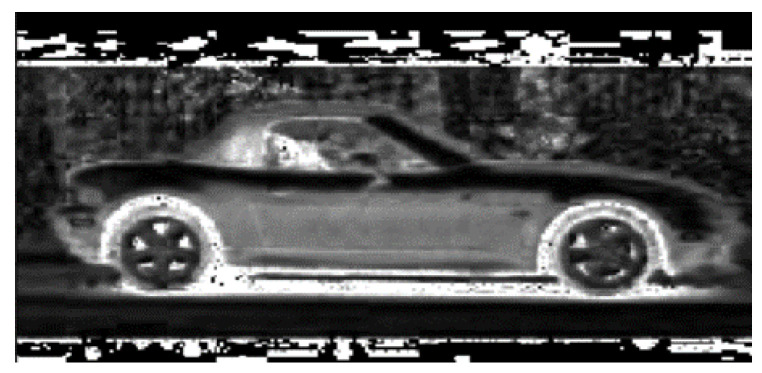
S.

**Figure 4:**
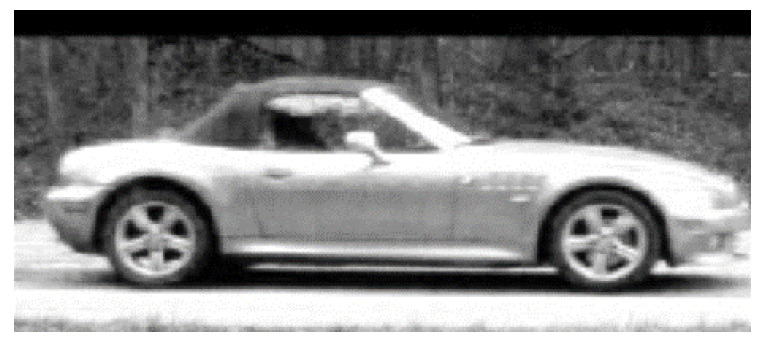
V.

The first alternate color space examined is HSV, where *H* represents hue, *S* saturation, and *V* value (often equated with brightness) as shown in Figure 2, Figure 3 and Figure 4. The separation of properties in the HSV color space provides an intuitive representation that closely mirrors human perception of color. More importantly for our purposes, with the effects of brightness isolated to the *V* channel, HSV may offer a degree of illumination invariance. It is anticipated that with HSV, re-identification may be more resilient to variations in a scene’s overall lighting. Additionally, training might benefit from the distinct color information provided by the hue, which is also isolated to its own channel in the HSV space.

The conversion to the HSV color space from RGB uses the following definitions.

(1) $$\begin{array}{cc} R^{\prime },G^{\prime },B^{\prime } & :\mathrm{RGB}\;\mathrm{values}\;\mathrm{normalized}\;\mathrm{to}\;[0,1]\\ M & =\max(R^{\prime },G^{\prime },B^{\prime })\\ m & =\min(R^{\prime },G^{\prime },B^{\prime })\\ C & =M-m \end{array}$$

where the chroma, *C*, is the difference between the maximum (*M*) and minimum (*m*) values among the *R*, *G*, and *B* components of color.

Hue (*H*) can be visualized as an angular position on the color wheel. It represents the type of color, such as red, blue, or green, and varies as one moves around the color wheel.

(2) $$\begin{array}{cc} H & =\left\{\begin{array}{cc} 0 & \mathrm{if}\;C=0\\ 60^{\circ }\times \left(\frac{G^{\prime }-B^{\prime }}{C}\;\mathrm{mod}\;6\right) & \mathrm{if}\;M=R^{\prime }\\ 60^{\circ }\times \left(\frac{B^{\prime }-R^{\prime }}{C}+2\right) & \mathrm{if}\;M=G^{\prime }\\ 60^{\circ }\times \left(\frac{R^{\prime }-G^{\prime }}{C}+4\right) & \mathrm{if}\;M=B^{\prime } \end{array}\right. \end{array}$$

To satisfy input expectations of the model, we calculate a normalized hue,

$$H^{\prime }=H/360^{\circ }$$

which, due to its dependence on quantized RGB values, lies in the half-open interval [0,1).

Saturation (*S*) represents the vividness of the color, where a saturation of 0 is grayscale (no chromatic content) and S=1 represents the purest form of the color.

(3) $$\begin{array}{cc} S & =\left\{\begin{array}{cc} 0 & \mathrm{if}\;M=0\\ \frac{C}{M} & \mathrm{otherwise} \end{array}\right. \end{array}$$

Value represents brightness, with 0 being black and V=1 being the brightest version of the color for the given *S*.

$$\begin{array}{cc} V & =M \end{array}$$

#### 2.1.3. YUV

**Figure 5:**
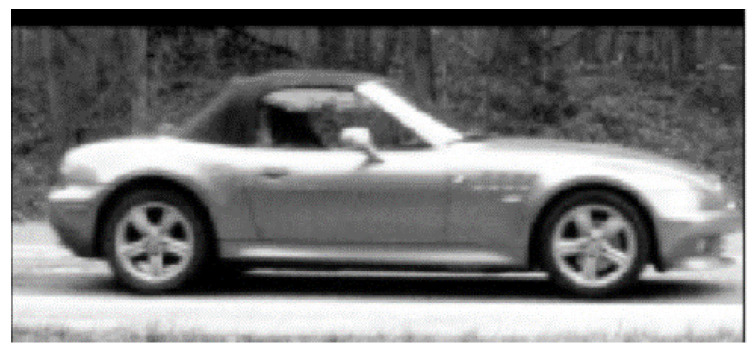
Y.

**Figure 6:**
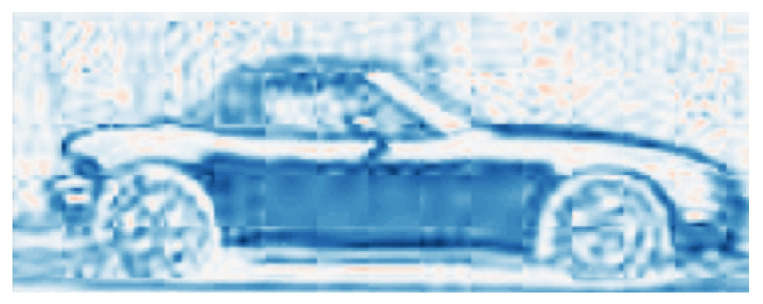
U.

**Figure 7:**
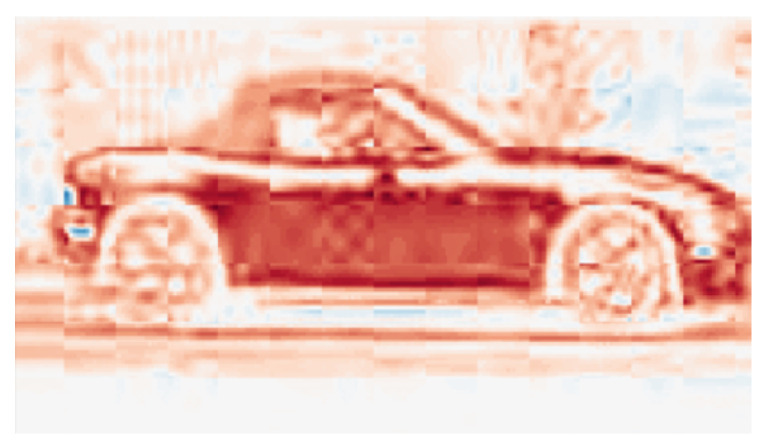
V.

The YUV color space [20,21] is potentially advantageous for applications in vehicle re-ID due to its separation of luminance (*Y*) and chrominance (*U* and *V*) components as shown in Figure 5, Figure 6 and Figure 7. This distinction mirrors human visual perception, where luminance carries significant information for recognizing the shapes and edges critical for identifying vehicles. With a channel dedicated to luminance, a network may better learn features relevant to vehicle shapes and structures that are stable under various lighting conditions and color variations. The 24-bit YUV configuration we use here preserves all the color information present in an original RGB image, thereby supporting discrimination among vehicles with similar structures but different color characteristics.

The cv2.cvtColor() function from the OpenCV library, with flag cv2.COLOR_RGB2YUV, implements the color space conversion as defined by the ITU-R Recommendation BT.601 standard [22]. The mapping is as follows:

(4) $$\begin{array}{cc} Y & =(0.299R+0.587G+0.114B)/255\\ U & =(-0.14713R-0.28886G+0.436B)/255+0.5\\ V & =(0.615R-0.51499G-0.10001B)/255+0.5 \end{array}$$

#### 2.1.4. LUV

The LUV color space emphasizes perceptual uniformity, closely aligning with differences in human color perception. Such alignment enables more accurate discrimination among vehicles of similar hues but different shades.

**Figure 8:**
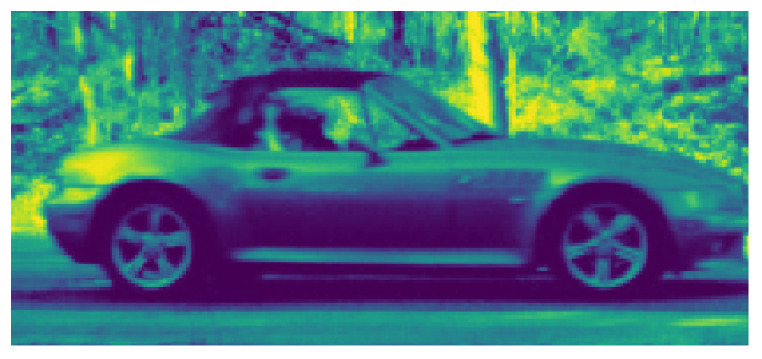
L.

The perceptual uniformity of LUV seems well suited for vehicle re-ID. Like YUV, LUV separates a luminance component ($L^{*}$) from chrominance components ($u^{*}$ and $v^{*}$). For the LUV chromaticity model, the separation supports a more nuanced representation of color variations, in principle enhancing the model’s ability to discern vehicles with subtle color differences. This feature reflects the importance of perceived color in vehicle identification, as manufacturers often choose colors to enhance visual appeal and distinctiveness, catering to human color perception. The *L* component is shown in Figure 8. Briefly, 24-bit mapping preserves the color information from the original RGB image in the LUV space.

We convert RGB to LUV using the OpenCV function cv2.COLOR_RGB2LUV. This conversion involves an initial transformation from RGB to the intermediate XYZ color space (where *Y* here represents luminance in the context of CIE 1931 color space), followed by a conversion from XYZ to LUV. The mapping adheres to the CIE 1976 $L^{*},u^{*},v^{*}$ standard with

(5) $$\begin{array}{cc} L^{*} & =116\;\cdot \;f(Y/Y_{n})-16\\ u^{*} & =13\;\cdot \;L^{*}\;\cdot \;\left(u^{\prime }-u_{n}^{\prime }\right)\\ v^{*} & =13\;\cdot \;L^{*}\;\cdot \;\left(v^{\prime }-v_{n}^{\prime }\right) \end{array}$$

where $f(Y/Y_{n})$ is a transformation applied to the normalized luminance, using a cube root function for higher luminance levels and a linear transformation for lower levels, in accordance with human visual perception. In the equations, $u^{\prime }$ and $v^{\prime }$ are chromaticity coordinates, and $u_{n}^{\prime }$ and $v_{n}^{\prime }$ represent the chromaticity coordinates of the reference white point, a standard reference in colorimetry for pure white.

#### 2.1.5. nRGB

Normalized RGB (nRGB) scales traditional RGB values by the sum S=R+G+B on a pixel level. The normalized values $R^{\prime },G^{\prime },B^{\prime }$ are as follows:

(6) $$\begin{array}{cc} R^{\prime } & =R/S\;,\\ G^{\prime } & =G/S\;,\\ B^{\prime } & =B/S\;. \end{array}$$

**Figure 9:**
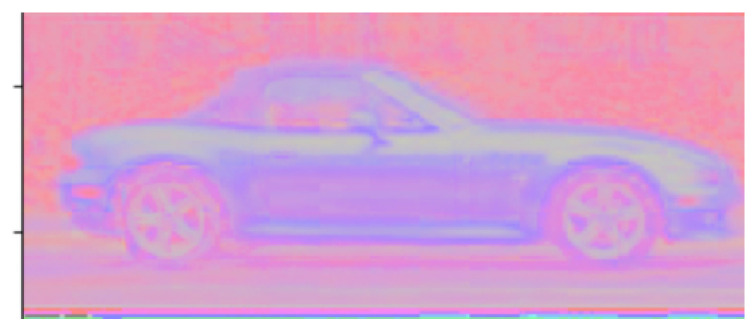
nRGB.

Advantages of nRGB include a level of invariance under changes in illumination. This insensitivity is beneficial in scenarios where relative color information is more important than overall brightness. Additionally, nRGB can enhance the contrast of colors, making certain features more discernible as shown in Figure 9. It also provides a consistent basis for comparing the color content of different images, as all pixels are evaluated based on their color proportions.

While nRGB offers various benefits, it also has its shortcomings. The most significant disadvantage is the loss of luminance information. Furthermore, the normalization process can amplify noise in pixels with low RGB values. Finally, special precautions must be taken for pixels with S=0 to avoid division by zero.

#### 2.1.6. c1c2c3

**Figure 10:**
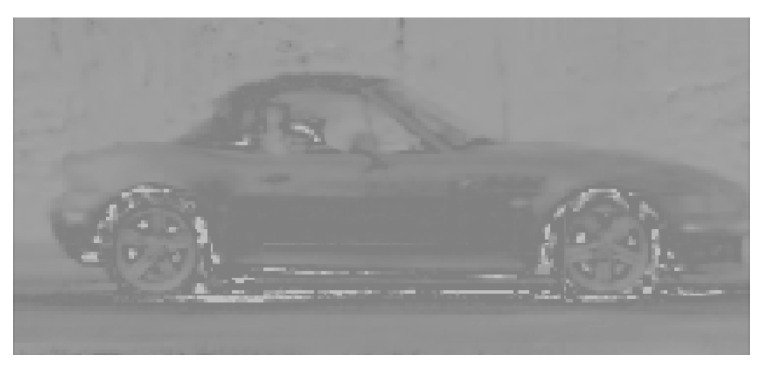
c1.

**Figure 11:**
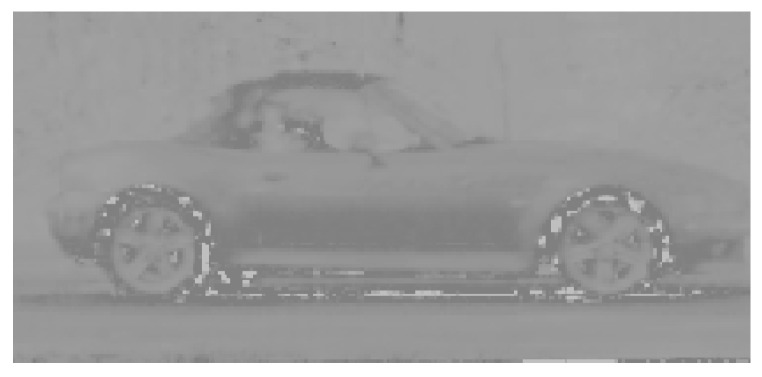
c2.

**Figure 12:**
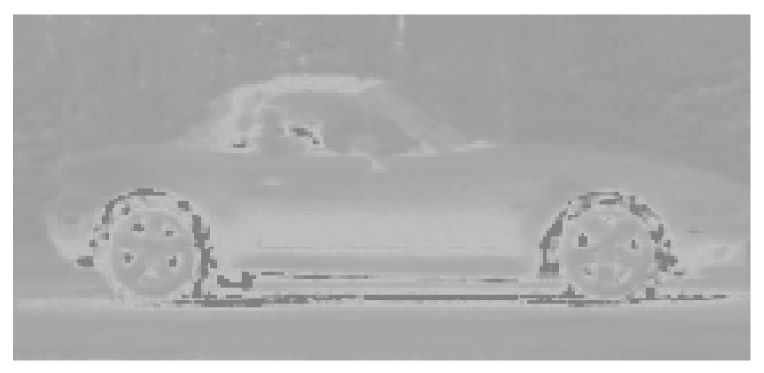
c3.

The c1c2c3 color space, introduced by Gevers and Smeulders, offers an alternative to the RGB color space that is potentially advantageous for visual re-identification [11]. It is resistant to lighting variations due to the use of the arctan function in the transformation from RGB as shown in Figure 10, Figure 11 and Figure 12. This function creates a bounded transformation that maps ratios of color channels to angles. The color representation becomes less sensitive to multiplicative changes in lighting, which uniformly alter the intensity across the image, because the angles—derived from the ratios of RGB color channels using the arctan function—remain constant under these uniform illumination adjustments. This approach aims to achieve illumination and shadow invariance while also providing highlight invariance by reducing the impact of specular reflections on color perception, ensuring consistent color representation even in the presence of bright light sources.

The definitions of the c1, c2, and c3 components within the c1c2c3 color space are as follows:

(7) $$\begin{array}{cc} c1 & =\arctan(R/G)/\pi +0.5\;,\\ c2 & =\arctan(R/B)/\pi +0.5\;,\\ c3 & =\arctan(G/B)/\pi +0.5\;. \end{array}$$

Here, the scaling and shifting of the output ensure that each channel, c1, c2, and c3, falls within the range [0,1] expected for input to the neural network.

#### 2.1.7. Twelve-Bit RGB and *n*-Bit Grayscale

**Figure 13:**
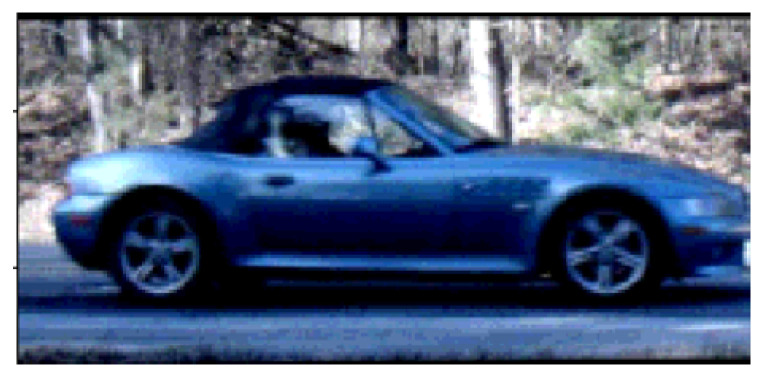
12-bit RGB.

Finally, we reduce the color resolution in RGB images to evaluate the impacts of this type of information loss on our vehicle re-identification model. The first experiment in color downsampling transforms standard 24-bit RGB imagery to 12-bit RGB imagery as shown in Figure 13. A second set of experiments converts 24-bit RGB images to *n*-bit grayscale ones, where *n* is a positive integer up to 8. These latter conversions limit pixel intensity levels to $2^{n}$ distinct values.

To create a 12-bit image, each color channel (R, G, B) is reduced to 4 bits from 8 bits. The 256 possible intensity levels of each 8-bit channel are grouped into 16 bins for each of the 4-bit channels:

(8) $$X_{4}=\left\lfloor X\;\cdot \;2^{-4}\right\rfloor \;.$$

Here, $\left\lfloor x\right\rfloor$ denotes the floor function, which rounds *x* down to its nearest integer. The symbol $X\equiv X_{8}$ represents an original 8-bit channel value, while $X_{n}$ denotes its new channel value with a reduced bit depth of *n*.

After the bit depth reduction, each pixel value is normalized to the maximum value of 15:

(9) $${\tilde{X}}_{4}=X_{4}/15\;.$$

**Figure 14:**
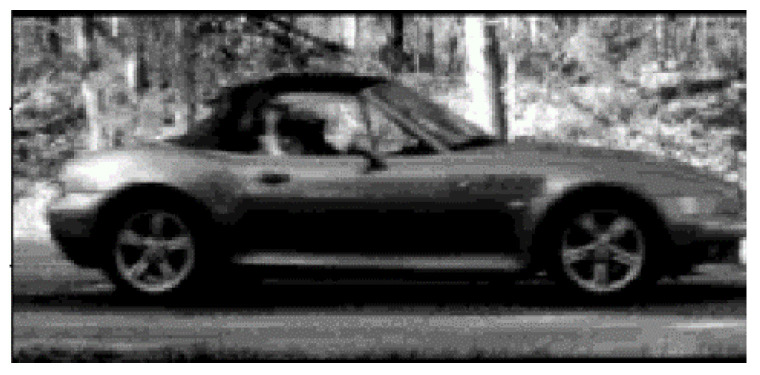
4-bit grayscale.

**Figure 15:**
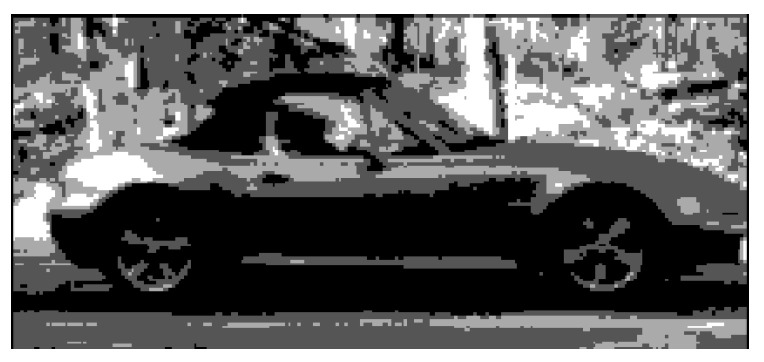
2-bit grayscale.

**Figure 16:**
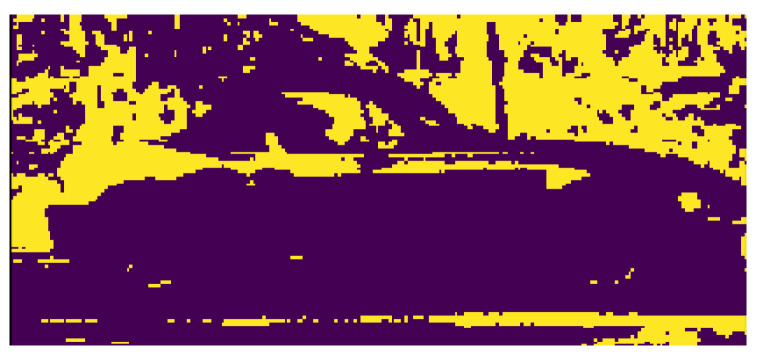
Binary.

Both the 12-bit RGB and *n*-bit grayscale conversions involve two core steps: a reduction in the number of distinct intensity levels and subsequent normalization. Continuing this thread, we consider the grayscale conversion, generalized to *n* bits, with our analysis variously taking *n* as a value in the set {8,4,2,1}. In Figure 14, Figure 15 and Figure 16, we show 4-bit grayscale, 2-bit grayscale and binary images respectively.

To perform the *n*-bit grayscale conversion, RGB images are first converted into grayscale using a weighted sum of the red, green, and blue channels with weights selected to represent human perception. We use the rgb2gray() function from the Python skimage.color module (version 0.22.0) to convert RGB values into grayscale as follows:

(10) Y=0.2125·R+0.7154·G+0.0721·B.

Similar to the 12-bit RGB method, reducing bit depth from an 8-bit grayscale image to an *n*-bit one involves limiting its intensity levels to $2^{n}$ distinct values. Grouping the 256 possible intensity levels of the 8-bit image into $2^{n}$ bins achieves such a reduction. To map these levels, each pixel value is divided by $2^{\left(8-n\right)}$ and then floored to its nearest integer, ensuring mapping to one of the $2^{n}$ possible new levels:

(11) $$Y_{n}=\left\lfloor Y\;\cdot \;2^{-(8-n)}\right\rfloor \;.$$

Normalization of $Y_{n}$ also proceeds in an manner analogous to that in the 12-bit RGB case. After a reduction in the bit depth, $Y_{n}$ lies in $\left[0,2^{n}-1\right]$. To prepare the image for input to the model, pixel values are normalized to the maximum value:

(12) $${\tilde{Y}}_{n}=\frac{Y_{n}}{2^{n}-1}\;.$$

Both methods decrease the information content of an image through bit depth reduction followed by normalization. The 12-bit RGB method retains some color differentiation, while the *n*-bit grayscale method replicates an image with reduced bit depth across the RGB input channels of the model. Our primary focus lies in evaluating the impact of these image conversions on model performance (Section 3); however, we note that these methods could also influence computational complexity.

### 2.2. Dataset

**Figure 17:**
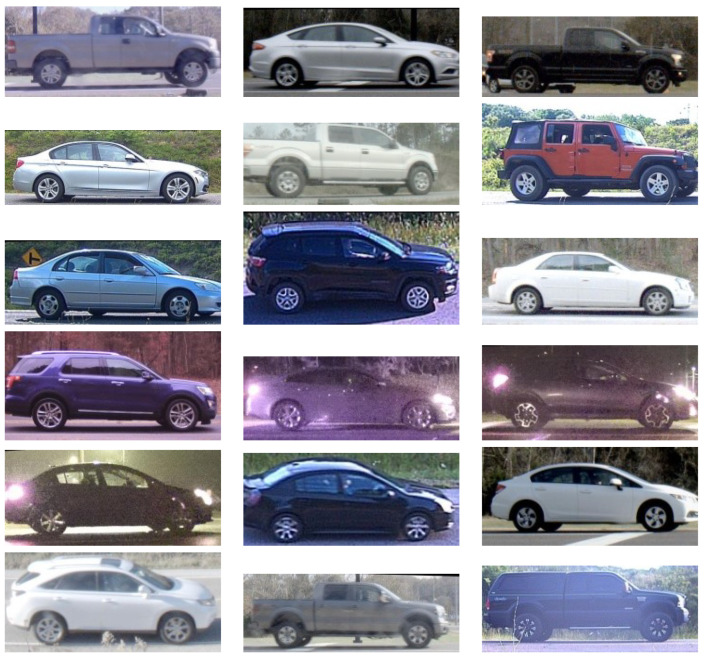
Sample images from the dataset.

Experiments use the Profile Images and Annotations for Vehicle Re-Identification Algorithms (PRIMAVERA) dataset [23], a comprehensive collection of side-view vehicular images employed in previous research on vehicle re-identification [24,25]. This dataset contains 636,246 images of 13,963 distinct vehicles, including daytime and night-time captures, collected over several years. For categorization of the images, the term daytime is defined as the period between local sunrise and sunset, whereas nighttime refers to the remainder of the day. Cameras equipped with 1.8–6 mm lenses were placed at various elevation angles and photographed passing vehicles from distances of 1–20 m. License plate readers established ground truth labels for the objects. Figure 17 displays select images illustrating the diversity of the dataset.

This study adopts the single-shot multibox detector (SSD) Mobilenet V2 network [26] for vehicle detection in video frames, following the approach in [24]. The SSD model, pre-trained on the Common Objects in Context (COCO) dataset [27], leverages the Caffe framework to place bounding boxes around vehicles in the frame. For this work, we retrained the SSD on a curated set of 543,926 images of 11,918 vehicles. Images were cropped and resized to a uniform 234×234×3, while preserving their original aspect ratios. They were flipped as necessary to standardize vehicle orientation, as depicted in Figure 17. Additionally, we trained a detector [24,25] to locate wheels, with a second retrained SSD instance providing coordinates of bounding boxes. To improve matching accuracy, we performed a pre-processing “wheel locking” step [24] to center front and rear wheels on the same two pixel locations in each image.

### 2.3. Network Structure

We train a matching network [24] on the dataset (Section 2.2) to compare a pair of vehicle images and produce a similarity score. The score serves as the basis for assessing whether input images represent the same object. The network employs a Siamese architecture [28] with two identical sub-networks that are updated during training with the same set of weights and biases across their corresponding layers. Siamese networks exhibit resilience against class imbalance and typically do not require retraining when a new class is introduced. In our matching network, a sub-network comprises seven layers with a max-pooling step following each layer. Outputs of the two branches are differenced, and a matching component with six layers produces a similarity score ranging from 0 to 1.

**Figure 18:**
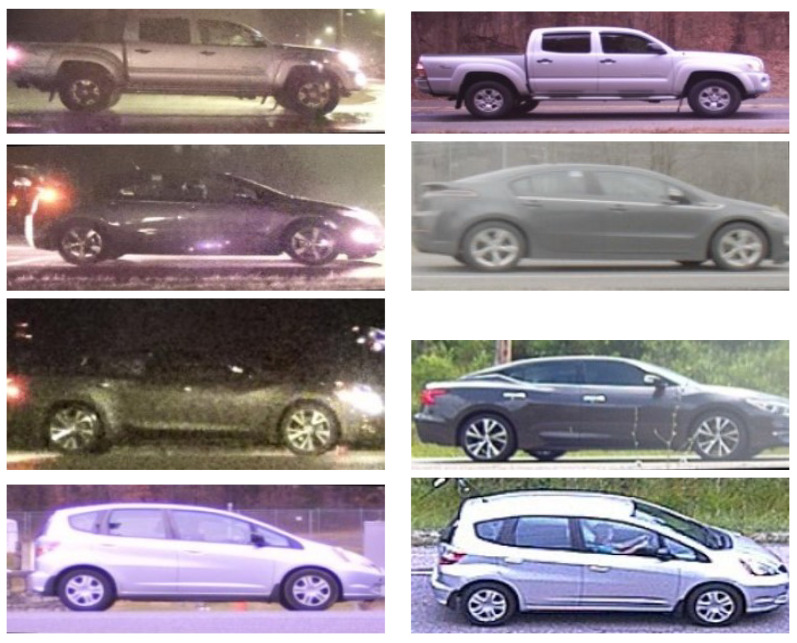
Example image pairs identified as true matches by the network.

**Figure 19:**
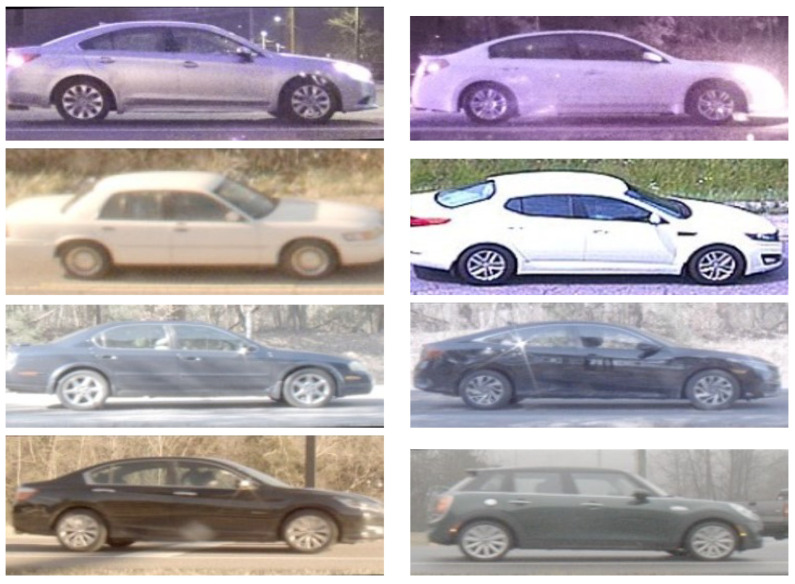
Example image pairs identified as false matches by the network.

For training and validation, we ensured diversity and consistency in the data by selecting random pairs of vehicular images from the dataset, with a fixed seed for the random generator across all color spaces. Hence, the sequences of pairs for training and validation were fixed across each color space that we considered in a given group. Because a group is defined by the subset of the dataset used in model development, the sequences vary by group, but within a group they do not. The selection included equal numbers of positive/negative, daytime/nighttime, and ground-level/elevated image pairs. We trained the network using the ADAM optimization technique [29] with a learning rate of 0.005, employing a binary cross-entropy loss function and a batch size of 256, representing 256 image pairs. This training continued for 100,000 iterations, with performance evaluated on a validation set containing 10,240 diverse pairs of vehicle images at 100-step intervals. The training performance was evaluated by averaging the accuracy over 100 batches at a time. Matching accuracy was determined using a threshold of 0.5 for the similarity scores, indicating a true match for scores above this threshold. Example images for true matches and false matches are depicted in Figure 18 and Figure 19, respectively.

## 3. Results

An experiment involves a two-step process: First, RGB images from the dataset (Section 2.2) are converted into another color space (Section 2.1). Following this conversion, an inference model is trained and validated on the transformed data. The outcome of the experiments is a series of predictions, each assessing whether a pair of vehicle images from the dataset represents the same object.

### 3.1. Model Accuracy

Accuracy, in the context of a binary classifier, is the fraction of model predictions that are correct. The number of correct predictions is the sum of true positives (TP) and true negatives (TN). The total number of predictions includes both correct and incorrect predictions:

(13) n=TP+TN+FP+FN

where FP and FN are the numbers of false positives and false negatives, respectively. The standard metric of accuracy, as the observed proportion of correct predictions,

(14) $$p=\frac{\mathrm{TP}+\mathrm{TN}}{n}\;,$$

ranges from 0 (completely inaccurate) to 1 (perfect accuracy) and provides a straightforward measure of a model’s overall correctness in its predictions. In Table 1, Table 2, Table 3 and Table 4, we depict the highest accuracy achieved during training and validation.

### 3.2. Color Space Performance

The effectiveness of vehicle re-identification models, measured through their accuracy, serves as a critical indicator of their potential for real-world applicability. To maintain consistency and ensure fair comparisons among models, the iterative training process starts by using the same randomly selected training and validation sets for all color spaces. Models based on data encoded in the native RGB color space of the cameras serve as a baseline for evaluating the use of alternate color spaces.

Models are categorized based on the subsets of the imagery dataset used in their development. Four categories are identified, and results are accordingly organized into distinct tables and associated figures. The *daytime* data, representing information collected during the day before sunset, were divided into training and validation sets, with the training set comprising 435,153 images and the validation set containing 76,203 images. The *nighttime* data, composed of images taken after sunset and before sunrise, were also split into training and validation sets, with the night-time training set containing 27,315 images, constituting around 6% of the total training data, and the night-time validation set comprising 4982 images. Experiments vary in their use of day/night data, including those that use day and night data in equal proportions (group I), those that use only one type of data (day only in II; night only in III), and those that utilize different types of data for distinct purposes (in group IV, selecting training data from all imagery, but validation data only from night-time collections). A given group consists of trials that assess the impact of each color mapping and change in color resolution in Section 2.1 on re-identification performance.

**Figure 20:**
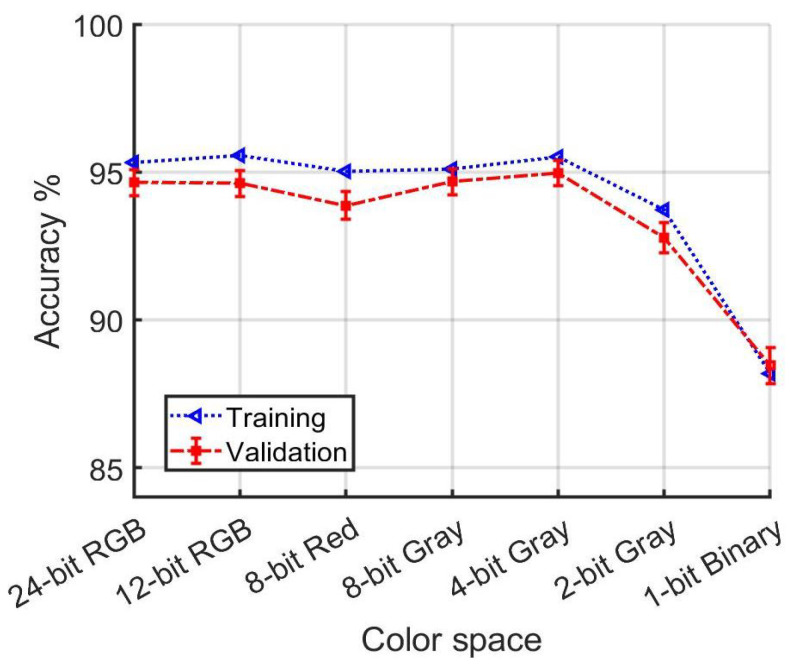
Table 1 detail. Accuracy dependence on color depth for models developed using both daytime and night-time data.

In the first group of training and validation runs, which selects examples from both daytime and night-time data, as shown in Table 1 and Figure 20, the YUV color space demonstrated superior performance over other color spaces for both training and validation under varied illumination conditions. Notably, 12-bit RGB performed comparably to 24-bit RGB, indicating a significant advantage even with reduced color depth. Contrary to expectations, no other transformations into 3D color spaces managed to surpass the performance of RGB. Remarkably, 4-bit Gray not only outperformed RGB but also achieved results that were, within the margin of error, comparable to those of YUV, underscoring its efficiency despite its reduced information content. Furthermore, even 2-bit Gray surpassed nRGB and c1c2c3 in terms of performance. It was only when the information content was reduced to a binary format that a noticeable drop in accuracy was observed.

**Figure 21:**
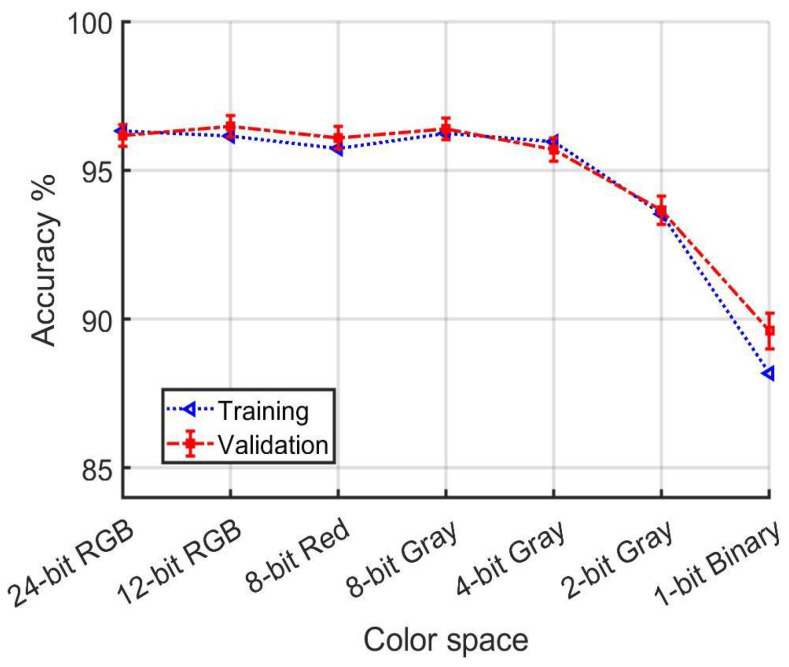
Table 2 detail. Accuracy dependence on color depth for models developed using only daytime data.

In the second set of experiments, which focused exclusively on daytime data for training and validation of the vehicle re-identification network, as detailed in Table 2 and Figure 21, the baseline RGB color space exhibited the top performance during the training phase. Interestingly, during validation, 12-bit RGB achieved the highest score overall, underscoring its effectiveness in conditions dominated by daylight. Furthermore, monochrome Red, Gray, 4-bit Gray, and 2-bit Gray also demonstrated commendable performance, indicating their utility even in the absence of diverse color information. This pattern suggests a nuanced relationship between color depth and recognition accuracy in vehicle re-identification tasks, especially under consistent lighting conditions.

**Figure 22:**
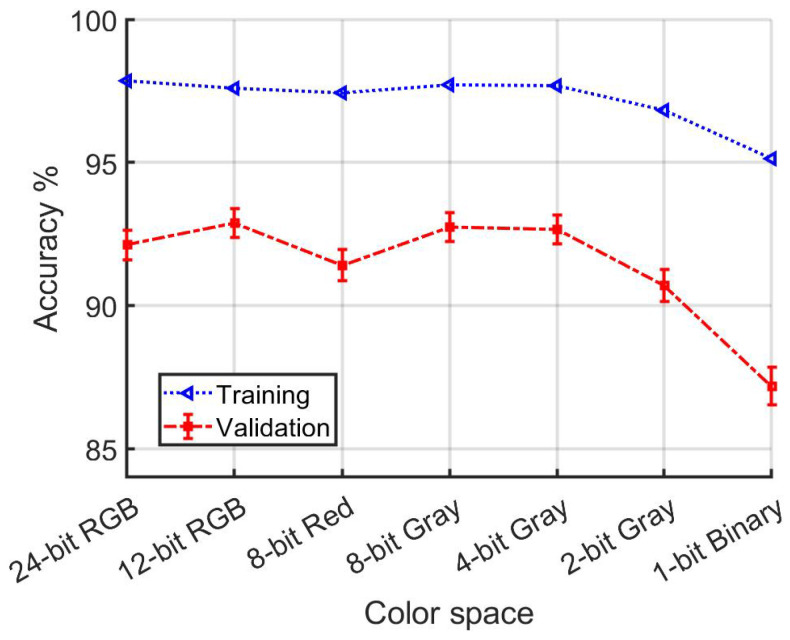
Table 3 detail. Accuracy dependence on color depth for models developed using only night-time data.

Table 3 and Figure 22 summarize the performance of models that use night-time data for training and validation. As in the second set of experiments, which used exclusively daytime data, the RGB color space achieved the best performance during training, while 12-bit RGB stood out during validation. Notably, a greater difference between training and validation accuracies was observed across all color spaces when compared with the daytime data results, indicating a pronounced challenge in model generalization under low-light conditions. Despite these variations, the disparity in reported accuracies across different color spaces, including Binary, was less pronounced for a given set of image pairs, whether during training or validation. However, nRGB and c1c2c3 consistently yielded the smallest accuracies during validation, highlighting the particular difficulties these color spaces face in accurately re-identifying vehicles with night-time imagery.

**Figure 23:**
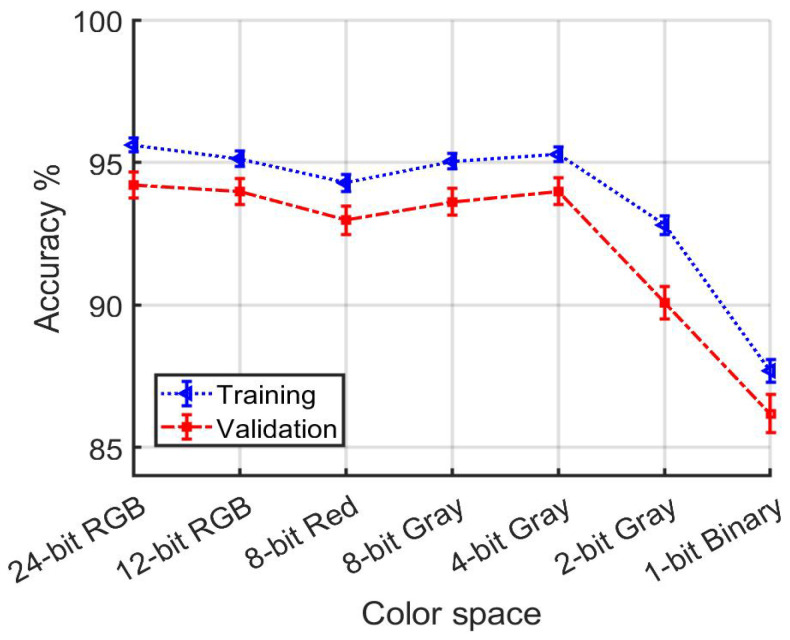
Table 4 detail. Accuracy dependence on color depth for models trained using daytime and night-time data and validated using night-time data.

Finally, Table 4 and Figure 23 cover experiments that explore the effects of training on all data and validating on night-time data. This adjustment to the training was made in response to the challenges observed in group III, which used exclusively night-time data. In group IV, by training the network with both daytime and night-time imagery, we sought to provide a more diversified learning experience and achieve better generalization when validating against the more challenging night-time data. RGB, HSV, and YUV were the standout performers in both training and validation phases, with HSV marginally being in the lead but within the margin of error, indicating closely competitive results among these color spaces. Consistent with findings from the other experimental groups, nRGB and c1c2c3 color spaces lagged behind, underscoring their relatively lower effectiveness in the specialized task of cross-illumination vehicle re-identification.

Table 1, Table 2, Table 3 and Table 4 show overlap among the accuracy confidence intervals from 24-bit color spaces down to the 4-bit Gray level for multiple training and verification datasets. Because model development is an inherently stochastic process, variation in accuracy is observed even when training is repeated using the same color space. Furthermore, this variation can supersede milder trends that are a function of color space. Loss of color resolution is only observed to materially degrade accuracy when we consider cases with less color resolution than 4-bit Gray.

**Table 1:** Accuracy of vehicle ID using both daytime and night-time data to train and validate the network.

	RGB	HSV	YUV	LUV	nRGB	c1c2c3	12-Bit RGB	8-Bit Red	8-Bit Gray	4-Bit Gray	2-Bit Gray	Binary
Training	95.32%	94.61%	**95.74%**	95.22%	92.80%	92.28%	95.56%	95.02%	95.10%	95.51%	93.72%	88.18%
Validation	94.65 ± 0.44%	93.75 ± 0.47%	**95.25 ± 0.41%**	94.51 ± 0.44%	91.95 ± 0.53%	90.05 ± 0.58%	94.62 ± 0.44%	93.87 ± 0.47%	94.67 ± 0.44%	94.97 ± 0.42%	92.78 ± 0.50%	88.45 ± 0.62%

**Table 2:** Accuracy of vehicle-ID using only daytime data to train and validate the network.

	RGB	HSV	YUV	LUV	nRGB	c1c2c3	12-Bit RGB	8-Bit Red	8-Bit Gray	4-Bit Gray	2-Bit Gray	Binary
Training	**96.32%**	95.87%	96.28%	96.14%	94.20%	94.27%	96.15%	95.73%	96.24%	95.96%	93.53%	88.18%
Validation	96.17 ± 0.37%	95.80 ± 0.39%	96.35 ± 0.36%	96.32 ± 0.37%	94.34 ± 0.45%	93.07 ± 0.49%	**96.48 ± 0.36%**	96.09 ± 0.38%	96.38 ± 0.36%	95.70 ± 0.39%	93.66 ± 0.47%	89.60 ± 0.59%

**Table 3:** Accuracy of vehicle-ID using only night-time data to train and validate the network.

	RGB	HSV	YUV	LUV	nRGB	c1c2c3	12-Bit RGB	8-Bit Red	8-Bit Gray	4-Bit Gray	2-Bit Gray	Binary
Training	**97.85%**	97.74%	97.75%	97.74%	96.97%	96.68%	97.59%	97.43%	97.71%	97.68%	96.82%	95.13%
Validation	92.11 ± 0.52%	92.31 ± 0.52%	91.90 ± 0.53%	91.93 ± 0.53%	86.93 ± 0.65%	85.09 ± 0.69%	**92.87 ± 0.50%**	91.41 ± 0.54%	92.74 ± 0.50%	92.66 ± 0.50%	90.70 ± 0.56%	87.18 ± 0.65%

**Table 4:** Accuracy of vehicle ID using daytime and night-time data to train the network but only night-time data for validation.

	RGB	HSV	YUV	LUV	nRGB	c1c2c3	12-Bit RGB	8-Bit Red	8-Bit Gray	4-Bit Gray	2-Bit Gray	Binary
Training	95.61%	**95.62%**	95.45%	95.21%	92.56%	91.86%	95.13%	94.28%	95.04%	95.28%	92.8%	87.69%
Validation	94.20 ± 0.45%	**95.09 ± 0.42%**	**95.09 ± 0.42%**	94.12 ± 0.46%	91.08 ± 0.55%	88.74 ± 0.61%	93.97 ± 0.46%	92.97 ± 0.50%	93.62 ± 0.47%	93.99 ± 0.46%	90.08 ± 0.58%	86.18 ± 0.67%

### 3.3. Confidence Intervals and Statistical Significance

The process of training and evaluating a model is inherently stochastic, driven by the unpredictable nature of the update step in stochastic gradient descent (SGD). Repeating the process therefore results in different values of model metrics. Because model development is resource-intensive, and because we explored twelve color spaces in combination with different subsets of the dataset, it was not possible to perform each evaluation multiple times. Nonetheless, it is straightforward to quantify confidence in the validation accuracy under reasonable assumptions.

We calculate a confidence interval, CI, for model accuracy during validation based on a normal approximation of the binomial distribution, for which we assume the following observations. Each observation is independent and identically distributed (i.i.d.), with each having the same probability of successfully determining whether or not a pair of images depicts the same vehicle. It is important to note that during a model’s training phase, ’observations’—or pair comparisons—cannot be considered to be drawn from the same distribution due to the ongoing development of the model. However, during the validation phase, the model is stable, making the i.i.d. assumption reasonable.

The confidence interval for the model accuracy, *p*, is as follows:

(15) $$\mathrm{CI}=Z\times \sqrt{\frac{p(1-p)}{n}}\;,$$

where *p* and *n* are as defined in (13) and (14), respectively. The central limit theorem is fundamental here, ensuring that the normal approximation is reliable given the large sample size in our experiments. Additionally, the expression for CI presupposes random sampling from the population and assumes that the number of trials, *n*, is fixed in advance, with no dependence on the outcomes of the observations. The Z-score is 1.96 for the 95% confidence interval we display for validation results in all tables and corresponding figures.

## 4. Analysis

General trends are apparent from a cursory inspection of the results. While color space and lighting conditions impact model performance, the effects are relatively modest. Only when the loss of color information approaches the point of producing a binary image is there a marked loss in accuracy.

### 4.1. Selection of Color Space

Evidence regarding the superiority of one color space over another for vehicle re-identification is inconclusive. Reported differences in accuracy among 24-bit color spaces are modest. No single color space consistently outperforms the others, though there is a marginal preference for YUV in scenarios where all data are utilized for both training and validation. Performance notably declines only when color content is significantly reduced, as seen when a model uses nRGB, c1c2c3, 2-bit Gray, or Binary features. Empirically, the models are often successful at distinguishing vehicles whose only apparent visually distinguishing feature is hue. Nevertheless, the sensitivity of validation accuracy to the choice of color space is at most marginally greater than the 95% confidence intervals. Ultimately, the incremental benefits do not strongly justify the additional processing required to convert from the native RGB format to another 24-bit color space.

The success of models in distinguishing between objects suggests a reliance on factors beyond color differences. In the absence of readily apparent alternatives, we hypothesize that geometrical features assume outsized importance in the re-identification process in our models. Geometry here refers not just to the projections of body contours onto the focal plane, which offer a silhouette or shape that can be distinctive from one vehicle to another, but also to the finer details observed in specific parts of the vehicles, such as the wheels. It is hard to know for certain what features a model keys on as it learns, but shape and structure, alongside color, would seem important for visual recognition here.

Validation accuracy closely matches training accuracy in the majority of cases, but a notable divergence occurs in experiments confined to night-time data (Table 3, Figure 22). This divergence indicates that the model may have become too closely attuned to the specific characteristics of the training data, failing to generalize effectively to new, unseen data in the validation set. The crux of the problem appears to lie in the relatively small size of the night-time dataset, which exacerbates the model’s tendency to overfit as it learns and relies on idiosyncrasies of the night-time data that do not represent broader data trends. This observation underscores the critical need for a balanced dataset to prevent overfitting and ensure that models retain their generalization capabilities across different lighting conditions. On the other hand, while color information is less pronounced in night-time imagery, its diminished presence alone does not account for the broader difficulties in generalization. This assertion is supported by the observation that deliberately choosing color spaces with limited color depth in daytime data does not significantly weaken the validation results compared with the training results in that case. Other factors, such as reduced visibility of vehicle contours at night, may play a larger role in the observed performance discrepancies relating to models trained on only night-time data.

### 4.2. Resilience against Loss of Color Information

A notable and perhaps surprising result is how gradually model performance degrades with reduced color resolution. Figure 20 shows that training and validation accuracy are comfortably above 90% even as color resolution is systematically reduced to 2 bits per pixel. Significant changes in performance become evident only when the information loss approaches the level of yielding a binary image. Indeed, the confidence intervals for accuracy using all data in Figure 20 overlap from 24-bit RGB to 4-bit Gray.

The resilience of model accuracy despite the loss of information aligns with the principles of the *curse of dimensionality* and its impact on feature selection and model simplification. This concept posits that in many cases, the true intrinsic dimensionality of data—essentially, the minimum number of features required for adequate representation—is significantly lower than the initially available feature set. In the context of our study, the vehicle images in our dataset possess a multitude of potential discriminative features. However, not all these features are equally critical for the model’s predictive capability. In our controlled campus environment, it emerges that a few key features, with color information being of limited importance, are paramount in influencing model decisions. By selectively altering the color information in image data inputs to a classifier, we engage in a form of implicit feature selection. This approach aids in narrowing down the input variables to identify those with the highest predictive value, demonstrating the importance of judicious feature selection in enhancing model efficiency and accuracy.

### 4.3. Implications for Vehicle Re-Identification

Given that model accuracy is only modestly affected by color space, the potential gains in processing efficiency from reducing color depth could be substantial and worthwhile. For example, converting imagery to 2-bit Gray would mean a 12-fold reduction in the amount of information to be processed. Such a reduction could potentially lower computational complexity, leading to quicker training and inference times beneficial across various applications. By focusing on the most critical features, we could also see improved model generalization and robustness, as the model becomes less prone to overfitting to noise or irrelevant details in the data. This streamlined approach could enhance deployment on resource-constrained edge devices, offering faster real-time processing and decision-making capabilities.

The vehicle re-identification system could be significantly simplified and streamlined by tailoring the visual collection equipment to directly capture images in 2-bit format. Equipment designed with this focus could be inherently simpler, lighter, and less expensive, as it would bypass the need for high-resolution sensors and the subsequent data reduction processing steps. This direct approach to data acquisition would not only reduce the computational load on the system but also enhance its adaptability and ease of deployment across various environments. The resulting system could offer enhanced utility in a wide array of applications, providing a cost-effective solution that maintains essential performance capabilities while leveraging the benefits of reduced data complexity.

Our results further suggest that dataset augmentation could enhance model accuracies. By increasing the number of example pairs with subtle color variations among similar vehicles, the model could be trained to more accurately distinguish vehicles based on color nuances. Although the study indicates that simplified data collection and processing might achieve comparable accuracies for broader applications, prioritizing color differentiation through dataset augmentation presents a promising avenue for applications where correctness is important. This approach not only underlines the potential for improving model accuracies but also highlights the adaptability of our methodologies to meet specific accuracy requirements in vehicle re-identification.

In an ideal vehicle re-identification scenario, each class would exclusively contain images of a single, specific vehicle. However, attaining this level of accuracy in a classifier system that omits license plate data is virtually impossible. This limitation results from vehicles of identical make, model, trim, and color typically lacking visible distinguishing features in their images. This inherent limitation directly restricts the maximum achievable accuracy in vehicle re-identification systems. Nonetheless, there is a positive aspect to consider. An inference model’s accuracy, when gauged in a controlled environment where the observed objects are drawn from a subpopulation, tends to be lower than the accuracy of that model when trained and used against a larger parent population. Consequently, we anticipate higher accuracy when the system currently under development is deployed against a larger pool of potentially observable vehicles.

## 5. Conclusions

In this paper, we explored the impact of various color spaces and color resolutions by identifying when degradation starts to represent a liability in the specific area of vehicle re-identification. Additionally, we examined the impact of environmental conditions on these findings. We trained and validated models for vehicle re-identification on a large dataset of images captured under diverse environmental conditions and categorized by day and night collection. The curated dataset was gathered within a controlled campus setting. The target color spaces in our study included diverse 24-bit representations of imagery as well as representations with progressively reduced color resolution, extending to the level of binary imagery. Conversions to these color spaces variously involved linear and nonlinear transformations.

We found that color spaces such as YUV, LUV, 12-bit RGB, 8-bit Gray, and 4-bit Gray are consistently competitive with RGB. Because conversion of imagery to 4-bit Gray did not appreciably affect accuracies, we infer that conversion to a lower-resolution space would streamline the analysis without sacrificing model performance. The potential gains in processing efficiency from reducing color depth are substantial. A reduction in resolution from RGB to 2-bit Gray would mean a 12-fold reduction in the amount of information to be processed, which would lower computational complexity, leading to quicker training and inference times that are beneficial across various applications. By focusing on the most critical features, we would also see improved model generalization and robustness, as the model becomes less prone to overfitting to noise or irrelevant details in the data.

Future investigations of the impacts of color space are planned using a multi-perspective vehicle re-identification system now under development.

## Data Availability

Profile Images and Annotations for Vehicle Reidentification Algorithms (PRIMAVERA). doi:10.13139/ORNLNCCS/1841347.
